# Assessing wild turkey productivity before and after a 14‐day delay in the start date of the spring hunting season in Tennessee

**DOI:** 10.1002/ece3.11390

**Published:** 2024-05-20

**Authors:** Joseph O. Quehl, Lindsey M. Phillips, Vincent M. Johnson, Craig A. Harper, Joseph D. Clark, Roger D. Shields, David A. Buehler

**Affiliations:** ^1^ University of Tennessee‐Knoxville Knoxville Tennessee USA; ^2^ University of Tennessee Knoxville Tennessee USA; ^3^ U.S. Geological Survey, Northern Rocky Mountain Science Center Knoxville Tennessee USA; ^4^ Tennessee Wildlife Resources Agency Nashville Tennessee USA; ^5^ Present address: Pheasants Forever Aitkin Minnesota USA; ^6^ Present address: West Virginia Division of Natural Resources South Charleston West Virginia USA

**Keywords:** hunting‐season framework, *Meleagris gallopavo*, regulation changes, reproduction, southeastern U.S., telemetry, wild turkey

## Abstract

Ten state wildlife management agencies in the United States, including six within the Southeast, have delayed their spring wild turkey (*Meleagris gallopavo*) hunting seasons since 2017 by five or more days to address concerns related to the potential effects of hunting on wild turkey seasonal productivity. One hypothesis posits that if the spring hunting season is too early, there may be insufficient time for males to breed hens before being harvested, thus leading to reduced seasonal productivity. We conducted an experiment to determine whether delaying the wild turkey hunting season by 2 weeks in south‐middle Tennessee would affect various reproductive rates. In 2021 and 2022, the Tennessee Fish and Wildlife Commission experimentally delayed the spring hunting season to open 14 days later than the traditional date (the Saturday closest to 1 April) in Giles, Lawrence, and Wayne counties. We monitored reproductive rates from 2017 to 2022 in these three counties as well as two adjacent counties, Bedford and Maury, that were not delayed. We used a Before‐After‐Control‐Impact design to analyze the proportion of hens nesting, clutch size, hatchability, nest success, poult survival and hen survival with linear mixed‐effect models and AIC model selection to detect relationships between the 14‐day delay and reproductive parameters. We detected no relationship (*p* > .05) between the 14‐day delay and any individual reproductive parameter. In addition, recruitment (hen poults per hen that survived until the next breeding season) was very low (<0.5) and did not increase because of the 14‐day delay. The traditional Tennessee start date had been in place since 1986 while the turkey harvest increased markedly until about 2006 and more recently stabilized. Our data indicate that moving the start of the hunting season from a period just prior to peak nest initiation to 2 weeks later, to coincide with a period just prior to peak nest incubation initiation, resulted in no change to productivity or populations in wild turkey flocks in south‐middle Tennessee.

## Graphical abstract

We assessed how a later spring wild turkey hunting season impacts wild turkey productivity to inform management agency regulation changes. To summarize, wild turkey productivity did not change if you allow the hunting season to open just prior to nest initiation (earlier in the year) or during the onset of nest incubation initiation (later in the year) in Tennessee.

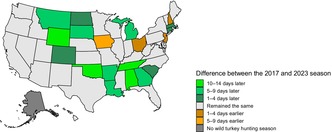

## 1 INTRODUCTION

The apparent decline in populations of wild turkeys (*Meleagris gallopavo*) is an important management issue in Tennessee and other states because turkey hunting is a popular activity and hunters prefer robust populations to provide quality hunting opportunities (Quehl et al., 2024). Many hunters and landowners have noticed declining observations of wild turkeys on their properties in portions of Tennessee (Shields, 2022), but causes for the perceived population declines are unknown and may differ from one area to another. Byrne et al. (2016) reported wild turkey productivity, as evidenced by poult per hen ratios, has been declining since 1990 in Tennessee and throughout the Southeast. Vanglider and Kurzejeski (1995) estimated >2.0 poults per hen in the fall were required to maintain a stable turkey population, and most states in the Southeast now are reporting ratios less than that. Agency biologists from Alabama, Georgia, Mississippi, North Carolina, and Tennessee reported 1.6, 1.5, 1.7, 1.3, and 1.4 poults per hen, respectively, for 2020 (Danks, 2021).

**FIGURE 1:**
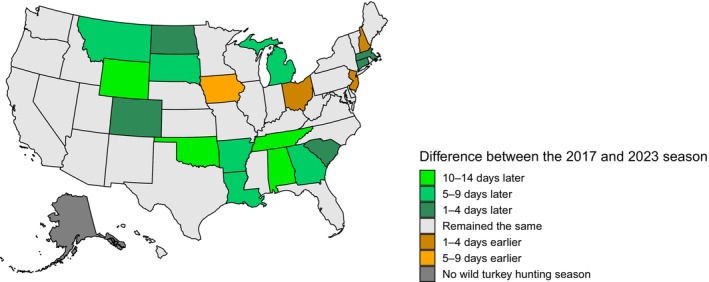
Differences in spring wild turkey hunting season start dates from the 2017 season to the 2023 season across all states in the U.S., grouped by: remained the same (no change), 1–4 days earlier or later in 2023 than 2017, 5–9 days, and 10–14 days. Sources for dates can be found in Quehl (2023).

Several hypotheses have been proposed to explain the decline in productivity and apparent population decline. These hypotheses include the effects of invasive species, such as feral pigs (*Sus scrofa*, Sanders et al., 2020) and armadillos (*Dasypus novemcinctus*), diseases associated with land management practices (Gerhold et al., 2016), changes in predator communities (Vander Haegen et al., 1988), density‐dependent population regulation (Byrne et al., 2016), and the timing of the spring wild turkey hunting season (Isabelle et al., 2018). The hypothesis related to the timing of the hunting season (hereafter referred to as “the later start date hypothesis”) has led six states in the Southeast (Alabama, Arkansas, Georgia, Louisiana, Oklahoma, and Tennessee) to delay the start of their spring hunting season 6–14 days since 2017 (Figure 1, see Quehl (2023) for sources).

The later start date hypothesis is based on two potential, nonmutually exclusive, mechanisms (Exum et al., 1987; Isabelle et al., 2016, 2018). First, if the spring hunting season starts too early, there may be a decrease in productivity if adult males are harvested before some hens are bred, and those hens do not nest. Second, wild turkeys establish a dominance hierarchy that correlates with breeding (Watts & Stokes, 1971). When a dominant male is removed, it may disrupt the hierarchy and interrupt breeding activity for an unknown period of time, potentially reducing the percentage of hens that are bred or the percentage of eggs that are fertilized. The disruption to the breeding cycle could delay or protract the breeding season, potentially decreasing productivity if earlier nests were more successful.

Although multiple states have delayed the spring hunting season to benefit reproductive success, there are no published data that support the later start date hypothesis. Whitaker et al. (2005) reported that the spring hunting season did not impact nesting phenology throughout the United States in hunted versus nonhunted populations, but they did not study the relationship between the timing of hunting season and nesting phenology. From 1986 through 2020, the spring hunting season in Tennessee has opened on the Saturday closest to 1 April and ended 44 days later. Median nest incubation date for initial nests in Tennessee is 27 April (Johnson et al., 2022) and median start of egg laying is 13 April. Therefore, the Tennessee hunting season generally begins before laying and well before the peak of laying and incubation. Since 1986, the turkey harvest in Tennessee increased markedly until 2006, when harvest began to oscillate and stabilize, typical of a population reaching carrying capacity (Del Monte‐Luna et al., 2004, Figure 2). Poults‐per‐hen ratios during that period in Tennessee, however, generally declined but have recently stabilized at <2 poults‐per‐hen (Byrne et al., 2016, Figure 2). Harvest has long been used as one of the main indices of wild turkey population growth; however, harvest is impacted by factors other than just population size, including hunter effort and regulation changes (Butler & Wang, 2022; Diefenbach et al., 2012).

**FIGURE 2:**
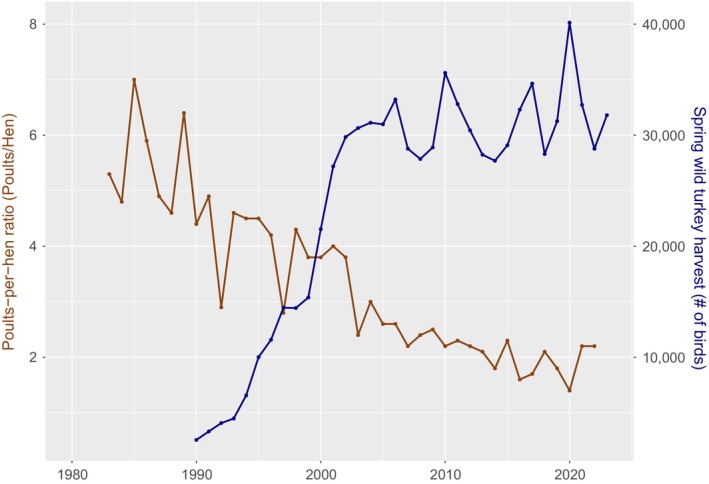
Statewide poults‐per‐hen ratio in Tennessee, USA, calculated from Tennessee's Wild Turkey Observation survey from 1983 to 2022 on the left *y*‐axis in brown, and statewide spring wild turkey harvest in Tennessee, USA, from 1990 to 2023 on the right *y*‐axis in blue. Statewide harvest data are reported from statewide required check‐in of wild turkeys during the spring hunting season. Data provided by the Tennessee Wildlife Resource Agency (2023).

**FIGURE 3:**
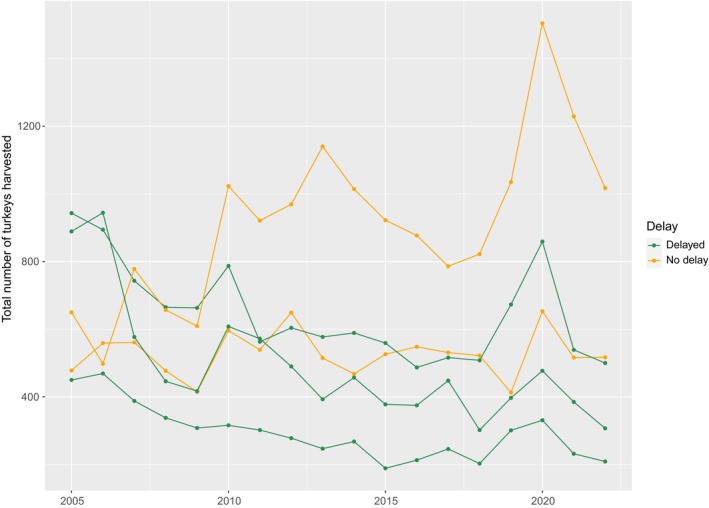
Annual spring harvest of wild turkeys in no‐delay counties (Bedford and Maury) and delayed counties (Giles, Lawrence, and Wayne) in south‐middle Tennessee, USA, 2005–2022. The delayed counties are believed to have declining populations of wild turkeys whereas the no‐delay counties are considered stable.

Although the statewide turkey harvest in Tennessee has stabilized in recent years, the harvest in some areas of the state has declined, especially in several counties in south‐middle Tennessee (Giles, Lawrence, Wayne), where harvest decreased by 60% from 2005 to 2015 (Figure 3, Tennessee Wildlife Resource Agency, 2023). Turkey hunters and managers are concerned about a decline in seasonal productivity and associated wild turkey abundance. To address the apparent population declines, the Tennessee Fish and Wildlife Commission voted to delay the opening date in 2021 and 2022 by 14 days in four counties with the greatest declines in spring harvest in Tennessee over the past 10 years (Figure 3).

Our objective was to assess wild turkey productivity in south‐middle Tennessee and evaluate whether the start date of the spring hunting season was related to productivity measures. We hypothesized that the start date could potentially influence nesting rate, nesting chronology, clutch size, hatchability, nest success, poult survival, and hen survival (Table 1). With additional time for turkeys to breed before reproductively active males could be harvested, we hypothesized that nesting rate and hatchability would increase and nesting would occur earlier in the spring. We also hypothesized that nest survival could increase in delayed counties because, with less disruption to the mating season (males being harvested prior to breeding), more hens may nest concurrently (i.e., predator swamping hypothesis/nesting synchrony; Ims, 1990; Robinson & Bider, 1988). Brooding wild turkeys generally select areas with herbaceous vegetation tall enough to conceal the brood, but not so tall to obscure the visibility of the hen (Healy, 1985; Nelson et al., 2023; Spears et al., 2007). Poult survival potentially could be lower with a later hunting season because if nesting is earlier, there could be less brood‐rearing cover available early in the growing season. We hypothesized that hen survival could increase because, if a larger portion of hens are incubating a nest during the hunting season, then they may be less likely to be harvested (Healy & Powell, 1999; Isabelle et al., 2018). We hypothesized that clutch size would be unaffected by the later hunting season because clutch size is determined primarily by intrinsic factors, such as genotypes or hen body condition, rather than extrinsic factors (Cody, 1966; Thogmartin & Johnson, 1999).

**TABLE 1:** Hypothesized effects of a 2‐week season delay on wild turkey productivity and survival parameters, south‐middle Tennessee, USA, 2017–2022.

Rank of influence	Parameter	Hypothesized effect after delayed hunting season	Justification
1	Median nest incubation initiation date (IID)	Earlier	Males have more time to breed, and dominant males are on the landscape longer so hens could initiate incubation earlier
2	Nesting rate	Increases	More time for males to breed with hens before potentially being harvested so more hens could initiate a nest
3	IID distribution	More contracted	Males have more time to breed, and dominant males will be on the landscape longer so hens may be bred and nest earlier and concurrently
4	Hatchability	Increases	Males have more time to breed, and dominant, reproductively active males are on the landscape longer, so hens could be bred more, which could lead to more fertilized eggs within the clutch
5	Daily nest survival/nest success	Increases	With less disruption to the breeding season, more nests may occur concurrently and experience greater nest survival
6	Daily poult survival/poult success	Decrease	Earlier nesting may lead to poults hatching earlier in the year. Poults on the landscape earlier in the year could result in poults having to use suboptimal vegetation cover and structure
7	Hen survival through nesting season—weekly estimates	Increases	Hen survival may increase because more hens are incubating nests while hunters are on the landscape, reducing the risk of illegal harvest and thus increasing their survival
8	Clutch size	Remains the same	Clutch size is predetermined based on genetics and hen health at the time of laying and less affected by external factors

## 2 STUDY AREA

**FIGURE 4:**
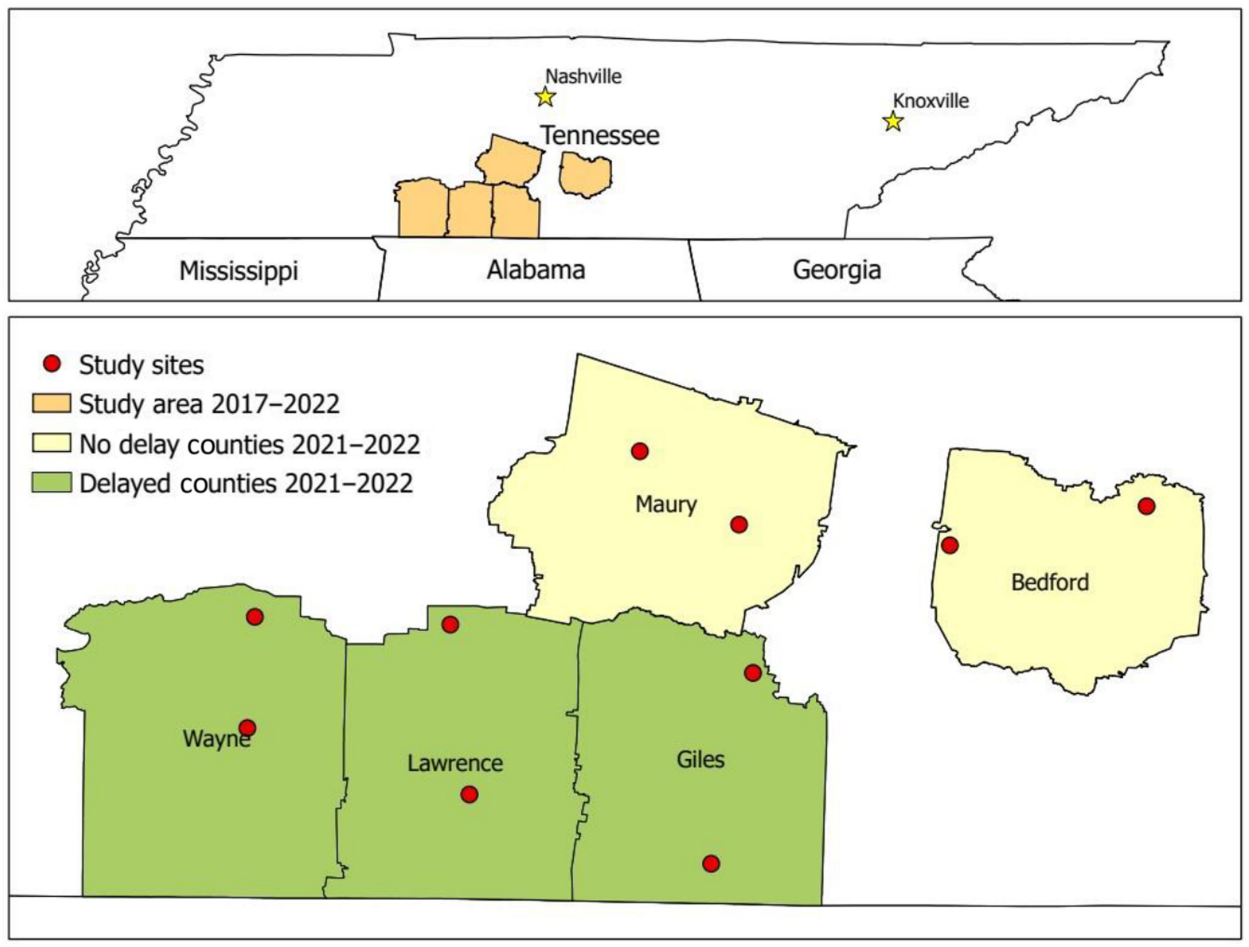
The five counties studied within south‐middle Tennessee, USA, with 10 trapping sites represented by red dots and counties separated by the start date of the spring wild turkey hunting season in 2021 and 2022.

We conducted our study in Bedford, Giles, Lawrence, Maury, and Wayne counties in south‐middle Tennessee, USA. We established two focal trapping sites strategically located in the northern and southern portions of each county where we had access to private and public lands for trapping and tracking radio‐tagged turkeys and for monitoring nesting and brood‐rearing activity (Figure 4). Private lands included deciduous forests, pasture, hay fields, coniferous forests dominated either by planted loblolly pine (*Pinus taeda*) or naturally occurring eastern redcedar (*Juniperus virginiana*), human development, row crops, young forests (deciduous or coniferous trees less than 10 years old), and early successional plant communities dominated by shade‐intolerant herbaceous plant species and colonizing woody species. Private lands throughout the 10 study sites totaled >29,000 ha and included >380 individual landowners. Our study areas included the Tie Camp Wildlife Management Area (WMA, 1325 ha) in Wayne County and Yanahli WMA (5200 ha) in Maury County, Tennessee, USA. Tie Camp WMA was managed by Bascom Southern Timber Company for timber production. Yanahli WMA was managed for white‐tailed deer (*Odocoileus virginianus*), wild turkey, and northern bobwhite (*Colinus virginianus*) through various management strategies. Tie Camp and Yanahli consisted of deciduous and coniferous forests, row crops, young forests, and early successional plant communities. All 10 study sites on public and private lands were hunted during the spring wild turkey hunting season from 2017 through 2022. The average temperature from April to August for our area (Lawrenceburg, TN) ranged from 15 to 28°C (average low to average high). The average annual rainfall was 145.8 cm and about 12.1 cm per month (U.S. Climate Data, 2023). Predominant soil types included Bodine cherty silt loam and gravelly silt, Gladeville rock outcrop, Ashwood, Brandon silt loam, Biffle gravelly silt loam, and Frankstone cherty silt loam (USDA, 2023).

## 3 METHODS

We trapped wild turkeys by deploying rocket net box sets (Delahunt et al., 2011). We baited trap sites with shelled corn and monitored trap sites with infrared‐triggered cameras (Moultrie: Model MCG‐13202, Birmingham, Alabama, USA). We checked and rebaited trap sites every 2–3 days. We also used cameras to monitor flock size, bait‐site visitation rates, and the age (adult vs. juvenile) and sex ratios of flocks visiting the trap sites. Our goal was to maintain a radio‐tagged sample of ≥10 hens (number of adults and juveniles was based on availability) at each focal trapping site each year.

We banded hens with uniquely numbered aluminum leg bands (National Band and Tag Company: style 1242FR8A, Newport, Kentucky, USA). From 2017 to 2018, we radio‐tagged all hens with very high frequency (VHF) transmitters (Advanced Telemetry Systems: Series A1500, Isanti, Minnesota, USA) via backpack harnesses (Guthrie et al., 2011). Beginning in 2019, we radio‐tagged about three hens per site with backpack global positioning system (GPS) transmitters (Lotek: GPS PinPoint, Wareham, the United Kingdom) and the rest with VHF transmitters. The VHF transmitters weighed ~80 g with a life expectancy of 5.7 years, whereas the Lotek GPS transmitters weighed ~92 g and had an expected battery life of 2.5 years. Actual GPS transmitter life was often less than 2 years. All transmitters were equipped with an 8‐hour mortality indicator switch. We released each bird at the trap site immediately after processing (University of Tennessee IACUC protocol #0561‐0720).

We monitored each radio‐tagged hen for movement, nesting activity, and survival. During the nonbreeding season each year (5 August–1 April), we downloaded locations of GPS‐transmitted hens weekly; GPS locations were collected at 9:00, 15:00, and 23:59 h (roost location) each day. We triangulated hens with VHF transmitters twice per week and monitored mortality. When a mortality occurred, we retrieved the transmitter and determined the cause of death when possible based on field sign. Beginning 1 April of each year, we located all hens every 2–3 days to monitor for nesting activity. GPS transmitters recorded hen locations every 2 h from 7:00 to 18:00 h and one roost location (23:59 h) every day. VHF transmitters were equipped with an activity switch (the radio signal pulse rate increased if the hen was moving), which aided in detection of incubation.

### 3.1 Nest monitoring

We confirmed a hen was nesting once the hen began incubating a nest. A GPS‐transmitted hen was deemed as incubating a nest when GPS locations formed a ~25‐m diameter cluster, and the cluster contained one roost location at the presumed nest site (Moscicki et al., 2023; Yeldell et al., 2017). Hens with VHF transmitters were deemed incubating when they had decreased movements and then were inactive based on the activity switch during one triangulation (Johnson et al., 2022; Miller et al., 1998; Thogmartin & Johnson, 1999; Vangilder et al., 1987). We walked a 30‐m radius circle around the nest of VHF‐transmitted hens to estimate the nest location. We monitored nests for incubation activity from a nearby (≥100 m away) observation point and checked every other day to determine if the hen was still incubating the nest. Nest incubation initiation date for VHF‐transmitted hens was the median date between the last location away from the nest site and the first inactive location at the nest site. For GPS‐transmitted hens, the nest incubation initiation date was the date of the first roost location at the presumed nest site. We estimated hatch date by adding 28 days to the nest incubation initiation date (Fuller et al., 2013; Spears et al., 2005). We monitored nests daily for 5 days prior to the estimated hatch date until the hen was no longer at the nest. If apparent incubation of a successful nest lasted >32 or <24 days, we adjusted the nest incubation initiation date to 28 days prior to the hatch date. Once the hen left the nest for >3 h and was >250 m away from the nest, we considered the nest no longer active (Hubbard et al., 1999a). We located the nest and determined nest fate (hatch or fail) based on the condition of the eggshells (Tyl et al., 2020). Once we located a nest, we recorded clutch size, number of hatched eggs (if applicable), GPS coordinates of the nest, nest vegetation, and a description of the nest.

### 3.2 Brood monitoring

We monitored broods by tracking radio‐tagged poults and conducting brood flush counts. We captured poults by hand after flushing the brooding hen before sunrise while ground roosting within 1–8 days post‐hatching (Hubbard et al., 1999b). All captured poults were placed in a cooler with a heating pad to keep them warm (Hubbard et al., 1999b; Spears et al., 2005). We radio‐tagged one to six poults within each captured brood in 2018–2022 by suturing the transmitter (Advanced Telemetry Systems: Series A1065, Isanti, Minnesota, USA) to their back (Burkepile et al., 2002; Johnson, 2019). The transmitters weighed 1.3 g and had a life expectancy of about 77 days based on field testing. We released captured poults in the vicinity of the hen at dawn to reunite the brood with the hen. Five poults in four broods apparently did not reunite with the hen (<3%) and were omitted from the analysis.

Each tagged poult was monitored for survival by homing and circling to within 30 m of the brood, similar to locating a nest (Hubbard et al., 1999b). While circling the hen and brood, we listened for the poult radio signals to determine if they were alive or dead. If the poult transmitters were located near the hen, we assumed the radio‐tagged poult was alive. If the poult radio signal was heard in the area but not associated with the hen, we homed to the transmitter to determine if the poult was dead. When a poult mortality occurred, the site was examined and a cause of death was determined based on field sign (Peoples et al., 1995; Speake et al., 1985). We considered a poult to be missing if the radio signal was not heard during the brood monitoring attempt. For the first 7 days post‐hatching, we monitored transmitted poults daily via circling. After day seven, transmitted poults were monitored every other day until day 28 post‐hatching. In addition to monitoring via telemetry, we flushed each brood on days 14 and 28 post‐hatching (Hubbard et al., 1999b; Peoples et al., 1995). We recorded the number of poults and hens present when flushed along with date, time, and GPS coordinates of the brood's location.

### 3.3 Data analysis

We monitored reproductive rates in the five focal counties for six consecutive years, 2017–2022, and analyzed the data in a Before‐After‐Control‐Impact study design (BACI, Smokorowski & Randall, 2017). Giles, Lawrence, and Wayne counties were considered impact or treatment counties affected by the season delay (hereafter, “delayed counties”), and Bedford and Maury counties were used as control counties (hereafter, “no‐delay counties”). We considered reproductive rates from 2017 to 2020 as before the season delay and rates from 2021 to 2022 as after the season delay.

We estimated the proportion of hens nesting, nest incubation initiation date (median and mean), clutch size, hatchability, daily nest survival, daily poult survival, and hen survival. We only included initial nesting attempts in these analyses, except for poult and hen survival, because the 2‐week delay coincided with the timing of initial nesting attempts. We assumed renesting was unaffected by the season opening date, which in some cases happened more than 2 months later. Hen survival was modeled across the entire nesting season because shifts in the timing of nesting could impact survival at various stages of the reproductive cycle. Nest failure during the laying stage may have resulted in missed nesting attempts. To account for this, we truncated the initial nesting period to 10 June of each year as this was the latest initial nest documented by our GPS‐transmitted hens.

We defined nesting rate (NR) as the proportion of hens that incubated a nest within a given year. Our nesting rate estimates are likely an underestimate of the true proportion of hens that attempted a nest each year (i.e., laid at least one egg) because some nests likely failed prior to documentation of incubation or failed during the egg laying phase. We calculated NR by dividing the number of hens that incubated a nest by the number of hens alive on 1 April of each year (Londe et al., 2023; Norman et al., 2001). Hens that died between 1 April and 1 May and were not documented incubating a nest were censored from this analysis as they may not have had sufficient opportunity to incubate a nest once the nesting season started (Thogmartin & Johnson, 1999). We defined nest incubation initiation date (IID) as the date the hen began incubating the nest. We used IID for initial nesting attempts to determine the mean and median date of nest incubation in each treatment before and after the season delay. We incorporated hen ID (unique identifier for each individual hen) as a random effect because some hens survived long enough for multiple nesting seasons throughout the study period. Timing of nesting distributions was analyzed using two, two‐sample Kolmogorov–Smirnov tests (delay‐before vs. no‐delay before, and delay‐after vs. no‐delay after) to assess changes in the distribution of IIDs. Nesting season length was calculated for three time periods: entire nesting season (first nest to begin incubation to last day of incubation for all nests); initial nesting time period (first nest to begin incubation to the last day of incubation for the last initial nest); and the renesting time period (first renest to begin incubation to the last day of incubation for the last renest). Time to renest was determined as the number of days from the initial nest attempt failing to the day the renesting hen began incubation. Clutch size (CS) was determined by counting the number of eggs found at the nest site. Hatchability (HABY) was the proportion of eggs within a nest to hatch (Londe et al., 2023). We only included hatched initial nests in the clutch size and hatchability analyses because the disturbance of depredated nests made it impossible to accurately determine the original number of eggs.

We used generalized linear mixed‐effect models to assess interactions between delayed and no‐delay counties before and after the season delay. We used a generalized linear mixed‐effect model with a quasibinomial error distribution to analyze nesting rate and hatchability. We chose the quasibinomial error distribution because nesting rates and hatchability are binomially distributed ratio data. We chose a Poisson error distribution for clutch size because these data were discrete counts. We analyzed nesting chronology using a linear mixed‐effect model that compared the ordinal date of IID for initial nests. Ordinal dates were box‐cox transformed (lambda = −2, y = ordinal date^−2^) to meet the normality assumption of linear models (Sakia, 1992). We analyzed all three periods for season length (entire nesting season, initial nesting time period, and renesting time period) using three general linear models. Shapiro–Wilk tests of normality were used to test the distribution of the data for the nesting season timing models outlined above. All models were created and analyzed in Program R (R Core Team, 2022). For all linear models, we adopted an *α*‐value of 0.05.

We calculated daily nest survival (initial nests), daily poult survival, and weekly hen survival through the nesting season using a staggered entry design (Pollock et al., 1989) in RMark (Laake, 2013). Daily nest survival (DNS) was defined as the probability of a nest surviving one day of the incubation period (Dinsmore et al., 2002). Daily poult survival (DPS) was the probability that a poult survived each day after hatching. Hen survival was calculated across the entire nesting season (1 April–5 August) because changes early in the nesting season from a 2‐week delay could potentially influence a hen's survival trajectory throughout the rest of the nesting season. We summarized hen survival into weekly survival intervals (Pollentier et al., 2014). We used 5 August as an end date for the nesting season because that was the last date a nest was known to have been incubated in any year of our study. We estimated survival using an information‐theoretic approach to evaluate potential relationships with covariates (Burnham & Anderson, 2002). We incorporated four covariates in our nest survival analysis: hen age, treatment (no delay vs. delayed) interacting with timing (before vs. after), year, and ordinal date of the nest incubation initiation date. These covariates resulted in 11 a‐priori models for daily nest survival. We then calculated nest success (NS) estimates by raising each daily nest survival estimate to the 28th power assuming a 28‐day incubation period (Londe et al., 2023).

We estimated poult survival with known‐fate models using survival data from radio‐tagged poults that hatched (Hubbard et al., 1999b). Seventy‐one radio‐tagged poults (38.7%) had unknown fates (i.e., went missing). We adjusted poult survival estimates to account for missing poults using 4‐week flush count data. We assumed a missing poult was dead on the first day they went missing if no poults were observed at the brood's 4‐week flush. Missing poults were censored after the first day the poult was not observed if ≥1 poult was observed at the brood's 4‐week flush. This method allowed us to account for any potential transmitter failure in our estimates. The poult survival analysis included the following covariates: hen age, treatment and timing interaction, year, ordinal date of the brood's hatch date, number of poults captured in a brood, and standardized mass at capture (mass/poults age). This analysis resulted in 13 a‐priori models that estimated daily poult survival. We raised daily poult survival estimates to the 28th power to estimate 28‐day poult survival (PS, Londe et al., 2023).

We divided hen survival during the nesting season into 18 weekly survival intervals that started 1 April of each year and ended 5 August. We used known‐fate models for this analysis, and we censored any individuals that went missing or dropped their transmitter. Covariates assessed in hen survival included age at the start of the nesting season, treatment and timing interaction, and year, which resulted in six a‐priori models.

For all survival analyses (nest, poult, and hen), the model we used to test the later start date hypothesis allowed survival to vary by treatment (delayed counties vs. no‐delay counties) and interact with timing (2017–2020 vs. 2021–2022) and will hereafter be referred to as the “interaction model.” We included additional models and covariates in our suite of models to test relevant hypotheses related to survival based on previous literature. We chose covariates to include in our models that may have been impacted by the season delay (i.e., nest incubation initiation date and number of poults produced) to help explain any differences that we observed in survival rates. Significant covariates were included in the interaction model to account for nuisance effects and variation.

To test for cumulative population‐scale effects of the season delay, we also estimated recruitment (R) for the entire nesting season. We defined recruitment as the number of female poults that are produced in a given breeding season that survive until the next breeding season per nesting female (Londe et al., 2023). For this analysis, we calculated renesting parameters (renesting rates, clutch size, hatchability, and nest success for renests) and survival of poults from 28 days post‐hatching to 365, hereafter referred to as youth survival ($S_{Y}$, Londe et al., 2023). We defined the renesting rate as the proportion of hens that failed an initial nesting attempt and attempted a second nest attempt. We censored hens that died within 30 days of the failed initial nesting attempt because they did not have sufficient time to renest (average time to renest = 24 days in our study area, Thogmartin & Johnson, 1999). We estimated youth survival based on equation (1) in Londe et al. (2023) to account for additional poult mortality observed after day 28 post‐hatching. For the annual survival rates, we used 1 April for the start of each year and summarized survival data into weekly survival intervals (Pollentier et al., 2014) and then analyzed in RMark (Laake, 2013). Equation (1) was adjusted to account for the weekly survival estimate:

(1) $$S_{Y}={S_{\text{Juvenile}}}^{\left(52-4\right)/52}$$

(Adjusted Equation 1; Londe et al., 2023).

We used equation (2) in Londe et al. (2023) to estimate recruitment per treatment (*c*) before and after the season delay (*t*):

$$R_{\mathrm{ct}}=\left[{\mathrm{NR}}_{1,\mathrm{ct}}\times {\mathrm{NS}}_{1,\mathrm{ct}}\times \frac{{\mathrm{CS}}_{1,\mathrm{ct}}}{2}\times {\text{HABY}}_{1,\mathrm{ct}}\times {\mathrm{PS}}_{\mathrm{ct}}\times S_{Y,\mathrm{ct}}\right]+\left[{\mathrm{NR}}_{1,\mathrm{ct}}\times \left(1-{\mathrm{NS}}_{1,\mathrm{ct}}\right)\times {\mathrm{NR}}_{2,\mathrm{ct}}\times {\mathrm{NS}}_{2,\mathrm{ct}}\times \frac{{\mathrm{CS}}_{2,\mathrm{ct}}}{2}\times {\text{HABY}}_{2,\mathrm{ct}}\times {\mathrm{PS}}_{\mathrm{ct}}\times S_{Y,\mathrm{ct}}\right]$$

Londe et al. (2023) estimated fecundity per age class (adult vs. juvenile) but in our analysis, we pooled all age classes, because of a low sample size of juvenile hens. We used the R package emdbook (Bolker, 2020) to calculate standard errors for fecundity based on the Delta method.

## 4 RESULTS

We captured 737 hens from 2017 to 2022, and radio‐tagged 432 with either a VHF (*n* = 283) or GPS (*n* = 149) transmitter. GPS‐transmitted hens accounted for 33% of radio‐tagged hens in no‐delay counties and 31% in delayed counties. Of the 737 hens captured, there were 609 adults and 115 juveniles, which resulted in 371 radio‐tagged adult and 61 radio‐tagged juvenile hens. The 432 radio‐tagged hens resulted in 623 hen‐years monitored for nesting activity and each hen was monitored for an average of 1.4 nesting seasons. We monitored 176 radio‐tagged hens in no‐delay counties and 256 radio‐tagged hens in delayed counties from 2017 to 2022, which resulted in 249 hen‐years in no‐delay counties and 374 hen‐years in delayed counties. We monitored 158 hen‐years from 2017 to 2020 and 91 hen‐years from 2021 to 2022 in no‐delay counties, and 242 hen‐years from 2017 to 2020 and 132 hen‐years from 2021 to 2022 in delayed counties.

### 4.1 Nesting parameters

Nesting rates in no‐delay counties were 0.74 (95% CI: 0.61, 0.86) and 0.85 (95% CI: 0.8, 0.89) before and after the season delay. In delayed counties, nesting rates averaged 0.71 (95% CI: 0.58, 0.84) before and 0.86 (95% CI: 0.78, 0.93) after the delay (Table 2). The generalized linear model showed no evidence of an interaction between nesting rate and treatment groups before and after the delay (*n* = 12, *β* = 0.20, SE_*β*_ = 0.90, *p*_Interaction, 11_ = .83, Table 3).

**TABLE 2:** Wild turkey reproductive rates measured from hens in south‐middle, Tennessee, USA, during 2017–2022, grouped by treatment and before and after the season delay. Estimates for nest success, poult survival, and hen survival were derived from interaction models with no additional covariates.

Reproductive rate	Treatment						Control					
	Before			After			Before			After		
	*n*	$\hat{y}$	SE	*n*	$\hat{y}$	SE	*n*	$\hat{y}$	SE	*n*	$\hat{y}$	SE
Nesting rate^a^	4	0.71	0.069	2	0.86	0.042	4	0.74	0.062	2	0.85	0.025
Median nest incubation date^a^	157	4/27	–	97	4/25	–	102	4/27	–	67	4/30	–
Nesting season length	4	110	1.548	2	111	10.5	4	101	2.345	2	103	8
Clutch size^a^	39	9.07	0.426	28	10.21	0.702	19	9.8	0.443	9	12.78	0.619
Hatchability^a^	34	0.84	0.031	27	0.87	0.036	18	0.91	0.038	9	0.85	0.09
Nest success^a^	149	0.287	0.036	90	0.349	0.051	97	0.204	0.038	66	0.191	0.044
Poult survival	47	0.16	0.054	78	0.156	0.040	34	0.052	0.029	24	0.268	0.098
Hen survival	229	0.725	0.030	125	0.762	0.039	149	0.708	0.037	84	0.688	0.051
Recruitment	–	0.108	0.046	–	0.200	0.078	–	0.031	0.020	–	0.112	0.090

^a^ Initial nests only.

**TABLE 3:** Summary of results from all interaction models used to assess the effect of the spring wild turkey hunting season start date in south‐middle Tennessee, USA, on eight reproductive rates of wild turkeys tested in 2017–2022 with associated models, *β*‐values, *p‐*values, and ΔAIC_c_ scores for each if applicable. Interaction models reported for nest success, poult survival, and hen survival are the highest ranked model that included the interaction term, Treatment × Timing, plus any additional covariate.

Reproductive rate	Interaction model formula	*β*	SE_*β*_	*p*	ΔAIC_c_	Effect of season delay
Nesting rate^a^	glm(NII ~ Treatment × Timing, family = Quasibinomial)	0.2028	0.8995	.83	–	No documented effect
Nesting season length	lm(SeasonLength ~ Treatment × Timing)	−1.75	9.116	.85	–	No documented effect
Nesting chronology^a^ ^,^ ^b^	lm(BC IID ~ Treatment × Timing + Age + (1|Hen ID))	0.00005	0.000007	.07	–	No documented effect
Clutch size^a^	glm(CS ~ Treatment × Timing, family = Poisson)	−0.154	0.1428	.28	–	No documented effect
Hatchability^a^	glm(HABY ~ Treatment × Timing, family = Binomial)	0.8215	0.8423	.33	–	No documented effect
Nest success^a^	S(~ Treatment × Timing)^28^	0.2252	0.2559	–	0	No documented effect
Poult survival	S(~ Treatment × Timing + Year)^28^	−0.6685	0.4335	–	1.37	No documented effect
Hen survival	S(~ Treatment × Timing + Hen Age)^18^	0.253	0.3382	–	6.945	No documented effect

^a^ Initial nests only.

^b^ Data were transformed using a box‐cox transformation with lambda = −2 (*y* = IID^−2^).

We evaluated nest chronology from 169 initial nests (102 before treatment, 67 after) in no‐delay counties and 254 nests (157 before treatment, 97 after) in delayed counties (423 total initial nests). Peak initiation of incubation occurred during the fourth week of April for all groups. Median nest incubation initiation dates were 27 April (first: 8 April, last: 30 May) in no‐delay counties and 27 April (first: 8 April, last: 5 June) in delayed counties before the season delay. After the delay, the median nest incubation date in no‐delay counties was 30 April (first: 14 April, last: 10 June) and 25 April (first: 6 April, last: 29 May) in delayed counties. Median nest incubation initiation dates varied by 5–12 days across years and treatment groups (Table 4). Our nest incubation initiation model showed a weak but insignificant relationship between season start date and nesting timing (*n* = 423, *β* = 0.000051, SE_*β*_ = 0.0000071, *p*_Interaction, 418_ = .07; Table 3). The model predicted a 2.8‐day shift later in no‐delay counties and 1.3‐day shift earlier in delayed counties for adult hens after the 2‐week delay. The juvenile hens shifted 3.2 days later in no‐delay counties and 1.5 days earlier in delayed counties. Age of incubating hen in this model was related to nest incubation initiation date, with adult hens nesting about 6 days earlier than juvenile hens (*β* = −0.0000063, SE_*β*_ = 0.0000026, *p*_Age, 418_ = .01). The distribution of IIDs were similar between treatment groups before the season delay (delayed‐before vs. no delay‐before, *p* = .22) and after the delay (delayed‐after vs. no delay‐after, *p* = .25).

**TABLE 4:** Table of yearly median nest incubation initiation dates for initial wild turkey nests in south‐middle Tennessee, USA, from 2017 to 2022 separated by treatment and hen age.

	Treatment			Control		
	Adult	Juvenile	All hens	Adult	Juvenile	All hens
2017	4/26	4/23^a^	4/26	4/25	4/25	4/25
2018	4/28	–	4/28	4/27	5/7^a^	4/27
2019^b^	4/28	–	4/28	4/20	–	4/20
2020	4/24	5/12^a^	4/24	4/29	4/19^a^	4/29
2021	4/23	4/24	4/23	4/26	5/1	4/28
2022	4/25	5/11^a^	4/28	5/2	5/19^a^	5/2

^a^ These estimates incorporate ≤3 initial nests.

^b^ No tagged juveniles nested in either county group.

The entire nesting season length before the season delay averaged 101 days (95% CI: 96, 106) in no‐delay counties and 110 days (95% CI: 107, 113) in delayed counties. After the season delay, the entire nesting season lasted 103 days (95% CI: 87, 119) in no‐delay counties and 111 days (95% CI: 90, 131) in delayed counties (Table 2). The initial nesting time period lasted 68 days (95% CI: 61, 76) and 78 days (95% CI: 70, 86) before the delay in no‐delay and delayed counties, respectively. After the delay, the initial nesting period lengthened to 72 days (95% CI: 47, 98) and 81 days (95% CI: 70, 91), respectively, in no‐delay and delayed counties. The renesting period lasted 77 days (95% CI: 74, 79) and 84 days (95% CI: 74, 95) before the season delay in no‐delay and delayed counties, respectively, then averaged 84 days (95% CI: 66, 102) and 86 days (47, 124) after the season delay in 2021 and 2022. The entire season‐length model showed no change in nesting season length that could be attributed to the season delay (*n* = 12, *β* = −1.75, SE_*β*_ = 9.11, *p*_Interaction, 8_ = .85, Table 3). The initial nesting time period (*n* = 12, *β* = −0.75, SE_*β*_ = 12.11, *p*_Interaction, 8_ = .95) and the length of renesting did not change in response to the delay (*n* = 12, *β* = −6.25, SE_*β*_ = 15.59, *p*_Interaction, 8_ = .70). Renesting began on 1 May for no‐delay counties and 2 May for delayed counties before the season delay and 4 May and 2 May, respectively, after the delay. Across all counties and years, the average time to renest was 24 days (95% CI: 22, 26).

We documented clutch size on 95 initial nests, including 58 nests from 2017 to 2020 (19 no‐delay, 39 delayed) and 37 nests from 2021 to 2022 (9 no‐delay, 28 delayed). The mean clutch size for initial nests was 9.8 (95% CI: 8.9, 10.7) and 9.1 (95% CI: 8.2, 10.0), respectively, in no‐delay and delayed counties before the delay. In 2021–2022, clutch sizes increased to 12.8 (95% CI: 11.6, 14.0) and 10.2 (95% CI: 8.8, 11.6) in no‐delay and delayed counties, respectively (Table 2). Based on the clutch size model with the interaction term, clutch size did not differ before or after the delay in the affected counties (*n* = 95, *β* = −0.15, SE_*β*_ = 0.14, *p*_Interaction, 91_ = .28, Table 3). Hatchability averaged 0.86 (95% CI: 0.82, 0.90) over all 6 years. Before the delay, hatchability was 0.91 (95% CI: 0.84, 0.99) in no‐delay counties and 0.84 (95% CI: 0.78, 0.9) in delayed counties. After the delay, hatchability was 0.85 (95% CI: 0.67, 1.00) in no‐delay counties and 0.87 (95% CI: 0.80, 0.94) in delayed counties (Table 2). The hatchability model indicated no difference before and after the season delay in delayed counties compared with no‐delay counties (*n* = 86, *β* = 0.82, SE_*β*_ = 0.84, *p*_Interaction, 82_ = .33, Table 3).

### 4.2 Survival estimates

We estimated daily nest survival using 402 initial nests, including 246 before the season delay and 156 after the delay, with 163 nests in no‐delay counties and 239 in delayed counties. Daily nest survival was 0.953 (constant survival, 95% CI: 0.947, 0.958) and equated to 0.254 (95% CI: 0.218, 0.299) nest success. The interaction model with no additional covariates had the most support of the 11 models analyzed and had a relative likelihood of 23% (*w* = 0.23, Table 5). However, confidence intervals for the *β* coefficient overlapped zero (*β* = 0.225; 95% CI: −0.276, 0.727; Table 3), indicating the relationship was not significant. Based on the interaction model, nest success was 0.204 (95% CI: 0.136, 0.283) before the delay and 0.194 (95% CI: 0.116, 0.289) after the delay in no‐delay counties. In delayed counties, nest success was 0.28 (95% CI: 0.212, 0.352) before the delay and 0.349 (95% CI: 0.253, 0.448) after. All other nest survival models ranked below the constant survival model (hereafter referred to as the “dot model,” Table 5). There was no support for the daily nest survival model, which included IID (*w* = 0.075, Table 5); indicating earlier initial nests were not more successful.

**TABLE 5:** AIC model results for daily nest survival with various covariates of initial wild turkey nests in south‐middle Tennessee, USA, from 2017 to 2022.

Model^a^	Number of parameters	AIC_c_	ΔAIC_c_	Weight	Deviance
S(~Treatment × Timing)	4	1902.775	0.000	0.230	1894.768
S(.)	1	1903.042	0.266	0.202	1901.041
S(~Treatment × Timing + IID × Year)	14	1904.043	1.268	0.122	1875.970
S(~Treatment × Timing + Hen age)	5	1904.486	1.711	0.098	1894.476
S(~Hen Age)	2	1904.636	1.861	0.091	1900.634
S(~Treatment × Timing + IID)	5	1904.777	2.002	0.085	1894.767
S(~IID)	2	1905.028	2.253	0.075	1901.026
S(~IID × Year)	12	1905.893	3.117	0.048	1881.838
S(~Treatment × Timing + Year)	8	1907.442	4.667	0.022	1891.417
S(~IID × Year + Hen age)	13	1907.706	4.931	0.020	1881.643
S(~Year)	6	1909.793	7.018	0.007	1897.779

^a^ Models allowed survival to vary by six covariates: treatment—dummy variable for delayed counties versus no‐delay counties, timing—dummy variable for nests in 2017–2020 or 2021–2022, nest incubation initiation date (IID), hen age (adult vs. juvenile), and year.

We radio‐tagged 183 poults from 2018 to 2022: 58 poults in no‐delay counties and 125 poults in delayed counties. We radio‐tagged 81 poults in 2018–2020 and 102 poults in 2021–2022. Of the 183 poults monitored, 33 poults survived 28 days post‐hatch (18.0%) and the fate of 71 poults were unknown during the 28‐day monitoring period (38.8%). Daily poult survival was 0.934 (95% CI: 0.921, 0.944) and 28‐day poult survival was 0.146 (95% CI: 0.101, 0.2). Yearly estimates of 28‐day poult survival ranged from 0.049 (2022: 95% CI: 0.01, 0.138) to 0.243 (2021: 95% CI: 0.15, 0.35). The interaction model to assess the impact of the season delay had a ΔAIC_c_ of 5.13 and a relative likelihood of 2.8% (*w* = 0.028, Table 6). The top model relating daily poult survival to year had a relative likelihood of 37.1% (*w* = 0.371, Table 6). This suggests poult survival is subject to significant annual variation. All other models not incorporating year had ΔAIC_c_ >2.0 and a relative likelihood of less than 3% (Table 6).

**TABLE 6:** AIC model results for daily poult survival estimates from radio‐tagged poults in south‐middle Tennessee, USA, 2018–2022.

Model^a^	Number of parameters	AIC_c_	ΔAIC_c_	Weight	Deviance
S(~Year)	5	901.215	0	0.37058	371.244
S(~Hatch date × Year)	10	901.827	0.61149	0.27296	881.707
S(~Treatment × Timing + Year)	7	902.585	1.37001	0.1868	368.586
S(~Treatment × Timing)	4	906.346	5.13049	0.0285	378.385
S(~Treatment × Timing + Hen age)	5	906.404	5.18929	0.02767	376.433
S(.)	1	906.895	5.68031	0.02165	384.955
S(~Hen age)	2	907.014	5.79862	0.0204	383.069
S(~Treatment × Timing + Hatch date)	5	907.192	5.97738	0.01866	897.16
S(~Treatment × Timing + Weight)	5	907.836	6.62135	0.01352	897.804
S(~Hatch date)	2	908.197	6.98152	0.01129	904.19
S(~Treatment × Timing + PT)	5	908.208	6.99323	0.01123	898.176
S(~Weight)	2	908.734	7.51857	0.00863	904.727
S(~PT)	2	908.862	7.64728	0.0081	904.856

^a^ Models allowed survival to vary by seven covariates: treatment—dummy variable for delayed counties versus no‐delay counties, timing—dummy variable for nests in 2017–2020 or 2021–2022, hatch date, hen age (adult vs. juvenile), year, poults trapped—number of poults caught in each brood, and weight—mass of the poult at the time of capture standardized by age of the poults.

We calculated weekly survival for 587 hens throughout the 2017–2022 nesting seasons. We monitored 149 hens before the season delay and 84 after the season delay in no‐delay counties. We monitored 229 hens before the season delay and 125 after the season delay in delayed counties. Weekly hen survival was 0.982 (95% CI: 0.979, 0.985) and hen nesting‐season survival (18 weeks) was 0.723 (95% CI: 0.685, 0.757). The top hen survival model included hen age (*β* = 0.741; 95% CI: −0.021, 1.502). Weekly adult hen survival was 0.982 (95% CI: 0.978, 0.984) and seasonal survival was 0.723 (95% CI: 0.671, 0.741). Weekly juvenile hen survival was 0.991 (95% CI: 0.981, 0.996) and seasonal survival was 0.849 (95% CI: 0.711, 0.925). The season delay interaction indicated no effect of the season delay as the *β* coefficients overlapped zero (ΔAIC_c_ = 6.945, *β* = 0.253, SE_*β*_ = 0.338, *w* = 0.021, Table 7).

**TABLE 7:** AIC model results for weekly hen survival throughout the nesting season of hens in south‐middle Tennessee, USA, during 2017–2022.

Model^a^	Number of parameters	AIC_c_	ΔAIC_c_	Weight	Deviance
S(~Hen age)	2	1604.49	0	0.672	700.971
S(.)	1	1607.06	2.565	0.186	705.537
S(~Treatment × Timing + Hen age)	5	1608.96	4.463	0.072	699.428
S(~Year)	6	1610.32	5.824	0.037	698.787
S(~Treatment × Timing)	4	1611.44	6.945	0.021	703.913
S(~Treatment × Timing + Year)	8	1612.65	8.155	0.011	697.112

^a^ Models allowed survival to vary by four covariates: treatment—dummy variable for delayed counties versus no‐delay counties, timing—dummy variable for nests in 2017–2020 or 2021–2022, hen age (adult vs. juvenile), and year.

### 4.3 Recruitment

Recruitment for no‐delay counties from 2017 to 2020 was 0.031 (95% CI: 0.00, 0.069) female poults produced per hen that survived to the next breeding season. Recruitment increased 264% in no‐delay counties to 0.112 (95% CI: 0.00, 0.285) in 2021 and 2022. Recruitment was 0.108 (95% CI: 0.02, 0.201) in delayed counties before the delay and increased 85% to 0.2 (95% CI: 0.048, 0.353) after the season delay.

## 5 DISCUSSION

Our models for all reproductive rates examined did not support the later start date hypothesis and showed no evidence that the later start date for the Tennessee spring hunting season impacted seasonal productivity. We saw no change in productivity in delayed counties, whether the hunting season began just prior to peak nest initiation (before the season delay) or just prior to peak nest incubation initiation (after the season delay). Based on the later‐start date hypothesis, the top two reproductive rates that we would have expected to change included the proportion of hens nesting (nesting rate), and hatchability (Table 1), neither of which were impacted by the start date of the spring hunting season. There was a weak relationship between nesting chronology and the season start date (*p*_Interaction, 418_ = .07), but this was represented by only 1 or 2 days in mean IID, which was well within the annual variation. This weak relationship lacks biological significance as we documented no net reproductive benefit in terms of greater success of initial nests earlier in the nesting season. Nesting rate and clutch size were greater in 2021 and 2022 after the 2‐week delay, but these increases occurred in both delay and no‐delay counties alike. Nest survival was unrelated to the season start date but we did observe higher nest success in Giles, Lawrence, and Wayne counties across all 6 years. Year‐to‐year variation in nest success averaged 5% in both delay and no‐delay counties and exceeded 10% some years. Such natural variation overwhelmed any potential difference in nest success associated with the 2‐week delay. Poult survival and hen survival were not impacted by the season start date as the interaction model had little support in both analyses. Importantly, there was no evidence to support the hypothesis that recruitment, based on estimates of hen poults produced per hen that survived until the next breeding season, increased as a result of the 2‐week delay.

### 5.1 Nesting parameters

The proportion of hens that attempt to nest should increase with a later hunting season if the hunting season is limiting reproductively active males from breeding. We found no evidence to support this expectation following a 2‐week delay of the Tennessee spring hunting season. Factors that influence yearly nesting rates are not well understood, but annual fluctuations are commonly observed within wild turkey populations (Vanglider & Kurzejeski, 1995). Changes in hen age ratios can influence nesting rates because juvenile hens nest at lower rates than adult hens (Vanglider & Kurzejeski, 1995).

Based on the later start date hypothesis, nesting chronology should have shifted earlier in delayed counties because of the additional time for males to breed. However, after 2 years of a 2‐week delay, the IID model did not demonstrate any biologically significant changes attributed to the season delay. Shifts in mean and median IID (1–2 days, and 2–3 days, respectively) before and after the delay were well within the annual variation in our study area prior to the delay (no‐delay: 9 days, delayed: 4 days; 2017–2020; Table 4). Median IID in the no‐delay counties varied by 12 days (2019 vs. 2022) over the course of the study. Variation in median IID was observed across treatment groups prior to the delay where median IID in no‐delay counties was earlier than delayed counties in 2017–2019, but later in 2020. In the second year of the season delay (2022), median IID in delayed counties was 28 April, which was the latest date for median IID in those counties across all 6 years (Table 4). Annual variation in nest incubation initiation could be influenced by annual variability in spring phenology or rainfall prior to nest initiation (Boone et al., 2023). The age of the hen was the only reliable predictor of nest incubation initiation date, which is consistent with the literature indicating adult hens initiate incubation earlier than juveniles (Londe et al., 2023; Norman et al., 2001; Quehl, 2023). Thus, annual variation in hen age ratios also could influence median IID.

Delaying the season start date to 15 April moved peak hunting pressure (the first week of the hunting season) into the early stages of incubation. However, nesting season length and time of nesting did not change in relation to the spring hunting season start date, which is inconsistent with the hypothesis that nesting would occur earlier in the year or that the distribution of nests over time would contract. None of our models indicated that nesting chronology (including median nest incubation initiation date, length, distribution, or renest timing) was impacted by the delayed season start date.

Our results supported our hypothesis that clutch size would be unaffected by the season delay. Clutch sizes were greater in no‐delay counties compared to delayed counties (*p*_Treatment, 91_ = .04) for unknown reasons, but this difference was observed in all years, not just after the season delay. Estimates of clutch size and hatchability for Tennessee were comparable to previous research in the eastern wild turkey's distribution (Davis et al., 1995; Pollentier et al., 2014; Thogmartin & Johnson, 1999; Tyl et al., 2020; Vanglider & Kurzejeski, 1995). There is no published data that indicates clutch size is affected by extrinsic factors but rather is influenced by genotype and hen body condition prior to egg laying (Cody, 1966; Lack, 1947; Thogmartin & Johnson, 1999).

Hatchability did not change in response to the season delay. Based on the later start date hypothesis, hatchability should increase because more reproductively active males are available with more time to breed hens and presumably increase egg fertilization. Our data indicate hens reproduced successfully prior to the delay when the hunting season opened just prior to peak nest initiation, and fertilization did not increase with a 2‐week delay. Although hatchability can be impacted by egg fertilization rates, other factors also can cause an egg to fail to hatch, such as early embryonic death (Birkhead et al., 2008). Current research investigating wild turkey egg fertilization may provide a better understanding of factors influencing hatchability (Gladkowski, 2023).

### 5.2 Survival estimates

Daily nest survival was not impacted by the season delay. The confidence interval of the *β*‐coefficient for the interaction model overlapped zero (*β*_Interaction_ = 0.225; 95% CI: −0.276, 0.727), and there was considerable within‐treatment variation (*β*_Treatment_ = −0.453; 95% CI: −0.848, −0.059). Average nest success in both no‐delay and delay counties varied as much as 10% from year to year prior to and after the delay. Giles, Lawrence, and Wayne counties (delay counties) had greater nest survival than Bedford and Maury counties (no‐delay counties) during all 6 years of the study regardless of season start date. Giles, Lawrence, and Wayne counties were the counties with the greatest decline in harvest in Tennessee from 2005 to 2015. Greater daily nest survival in those counties indicates that density dependence may be influencing the population and sites with lesser hen densities now have greater nest success (Byrne et al., 2016). Our nest success estimates (*S* = 0.25) were remarkably similar to estimates from other declining populations in the Southeast (0.26 Georgia, Bakner et al., 2019; 0.24 South Carolina, Lohr et al., 2020; and 0.24 Louisiana, Crawford et al., 2021). In 2023, the Tennessee Fish and Wildlife Commission implemented a 2‐week delay statewide such that all five counties in our study received the 2‐week delay treatment. Nest success in 2023 was poor across the five counties (*n* = 49 nests, *S* = 0.176, SE = 0.053), providing further evidence that the 2‐week delay did not improve nest success.

Ordinal date of nest incubation initiation, incubating hen age, and year received no support in the nest survival models, contrary to Keever et al. (2023), who reported nests earlier in the year were four times more likely to hatch than nests later in the year. However, Keever et al. (2023) included all nests (initial and all subsequent renests) in their analysis and did not report the effect of timing on survival of initial nests. We suggest that only initial nests are relevant for assessing the effects of the timing of the spring turkey season. The number of days from initial nest abandonment/depredation to onset of the first renest in our study varied from 5 to 64 days. Previous research has reported large yearly fluctuations in daily nest survival and nest success (Roberts & Porter, 1998), but year was among the lowest‐ranked covariates in our study (Table 5). We also observed no difference in daily nest survival between nests incubated by adults vs. juveniles, contrary to Norman et al. (2001), who reported juveniles had less reproductive success than adults.

We predicted that poult survival would decrease following the later hunting season start date because earlier nests could produce poults before adequate brooding cover and food were available. However, our results did not support this hypothesis as we saw no change in delayed counties (PS Before: 0.16 and after: 0.156) but saw an increase in no‐delay counties (PS Before: 0.052, and after: 0.268). The interaction model's *β* coefficients did not overlap zero (*β* = −0.851, 95% CI: −1.677, −0.025) but this interaction did not affect the delayed counties suggesting no effect of the season delay and rather just year‐to‐year variation. Poult survival averaged across the 6 years of the study was only 0.146 for the 28‐day interval. Few contemporary survival estimates based on monitoring radio‐tagged poults have been published but radio‐tagged survival estimates from the 1990s were greater then (0.24 New York: Roberts et al., 1995, 0.42 Iowa: Hubbard et al., 1999b). Survival estimates based on flush counts from various locations in the U.S were generally greater than our radio‐tagged estimates (0.255 Mississippi, Miller et al., 1998; 0.27 South Dakota, Thompson, 2003; 0.34 Wisconsin, Pollentier et al., 2014; 0.35 Texas, Isabelle et al., 2016; and 0.36 Georgia and South Carolina, Chamberlain et al., 2020), though method‐based biases likely exist.

We predicted that hen survival would increase following the season delay because more hens would be incubating during the first couple of weeks of the hunting season. Incubating hens have greatly reduced daily movements (Healy & Powell, 1999) and therefore are less susceptible to accidental harvest by hunters (Healy & Powell, 1999; Isabelle et al., 2018). However, the interaction model for hen survival through the nesting season was among the least‐supported models in the model set. In delayed counties, the hunting season start date following the delay (15 April and 16 April) was more closely aligned with peak incubation initiation (21 April), but we documented no changes in hen survival. During the 6 years of our study, none of our radio‐tagged hens were killed by hunters, including 16 bearded hens. Given these data, direct hunter‐based mortality did not affect hen survival in south‐middle Tennessee. Considering the extent of our study, including two public hunting areas and >380 individual private landowners, we interpret our results to be representative of turkey hunters at least throughout the middle Tennessee region.

### 5.3 Recruitment

We did not see a change in recruitment that we could attribute to the season delay as both treatment and control county groups were low initially in 2017–2020 and increased significantly in 2021 and 2022 (264% increase in no‐delay counties and 85% increase in delayed counties). Our estimates of recruitment were reflective of relatively poor nest survival and very poor poult survival, regardless of the 2‐week delay. Our 6‐year average estimates are lower than Londe et al.'s (2023) relatively low estimates for adults (0.34) and more comparable to the juvenile hen estimates of recruitment (0.18). Our estimates of recruitment were representative of the population because we radio‐tagged hens opportunistically at capture regardless of age. Our radio‐tagged sample of hens, however, was ~90% adults because we did not capture very many juveniles, consistent with relatively poor recruitment (*n* = 61 transmitted from 2017 to 2022).

Our results highlight the importance of a strong experimental design, Before‐After‐Control‐Impact. The BACI study design allowed us to directly compare a number of covariates by contrasting before and after effects of a treatment with before and after effects in areas without the treatment. We had a large sample size (>100 hens monitored per year) with 6 years of monitoring. However, our estimates of the interaction term (*β*) had wide CIs, which were driven by high natural annual variation. We conclude that natural variation had a greater impact on survival and recruitment parameters than any effect of the 2‐week delay. Based on the models for each reproductive rate (nesting rate, nest incubation initiation date [IID], nesting season length, IID distribution, clutch size, hatchability, nest success, poult survival, hen survival, and recruitment), we observed no changes in productivity to support the later start date hypothesis.

## 6 MANAGEMENT IMPLICATIONS

Assumptions implicit in the setting of spring turkey hunting season frameworks are that hunting does not disrupt reproductive behavior and does not affect long‐term population growth (Healy & Powell, 1999). Our data do not support the hypothesis that delaying the start date of the spring hunting season from just prior to peak nest initiation to just prior to peak nest incubation initiation would increase wild turkey productivity and ultimately increase recruitment into the population. We documented no effect of the 2‐week delay on wild turkey productivity, poult survival, hen survival, or recruitment. Our results did not demonstrate that beginning the wild turkey hunting season during the early stages of nest initiation disrupted the nesting process and decreased productivity when compared to beginning the season closer to the onset of incubation. In 2023, the Tennessee Fish and Wildlife Commission delayed the spring wild turkey hunting season 2 weeks later for all counties in Tennessee. Returning the opening date of the spring hunting season to early April will provide hunters with more opportunities to hunt birds when they are actively gobbling (Chamberlain et al., 2018; Quehl et al., 2024). Furthermore, feedback received from hunters indicated that satisfaction may decrease as hunters become aware that results of the study did not document an increase in productivity from delaying the opening of the spring turkey season (Quehl et al., 2024). We stress that we are not suggesting that timing or the harvest level of the spring hunting season cannot have an effect on wild turkey productivity and populations, but that timing the start of the spring turkey hunting season just prior to peak nest initiation and the harvest of ~30% of adult males per year did not negatively affect wild turkey productivity or populations on our study sites in Tennessee. To better understand ecosystem response outside our study area, state agencies could conduct similar research to determine the effect of a delayed season on wild turkey productivity prior to making hunting‐season framework changes.

## AUTHOR CONTRIBUTIONS

**Joseph O. Quehl:** Data curation (equal); formal analysis (lead); writing – original draft (lead); writing – review and editing (equal). **Lindsey M. Phillips:** Data curation (equal). **Vincent M. Johnson:** Data curation (equal); methodology (equal). **Craig A. Harper:** Conceptualization (equal); funding acquisition (equal); supervision (equal); writing – review and editing (equal). **Joseph D. Clark:** Formal analysis (supporting); writing – review and editing (equal). **Roger D. Shields:** Funding acquisition (supporting); writing ‐ review and editing (supporting). **David A. Buehler:** Conceptualization (equal); funding acquisition (equal); supervision (equal); writing – review and editing (equal).

## FUNDING INFORMATION

We received funding from the Tennessee Wildlife Resources Agency, University of Tennessee's School of Natural Resources, the National Wild Turkey Federation, and Turkeys for Tomorrow.

## CONFLICT OF INTEREST STATEMENT

The authors declare no conflicts of interest.

## Data Availability

These data are available for review at https://datadryad.org/stash/share/N5eD8fQRl6JMUQLRULMWZUX_Y2GhsQIyyyGzk9jct2w.
